# Excessive body weight of children and adolescents in the spotlight of their parents’ overweight and obesity, physical activity, and screen time

**DOI:** 10.1007/s00038-020-01419-x

**Published:** 2020-07-01

**Authors:** Erik Sigmund, Dagmar Sigmundová, Petr Badura

**Affiliations:** Faculty of Physical Culture, Palacký University Olomouc, Tr. Miru 117, 77111 Olomouc, Czech Republic

**Keywords:** Family, Overweight/obesity, Step counts, Organized leisure-time physical activity, Parent–child dyads, Nuclear family triads

## Abstract

**Objectives:**

The main aim of this study was to bridge the research gap in the countries of Central Europe using the family dyad approach to examine the associations of parents’ overweight/obesity, physical activity (PA), and screen time (ST) with excessive body weight in their offspring.

**Methods:**

The cross-sectional study included 1101 parent–child dyads (648/453 mother/father–child aged 4–16) selected by two-stage stratified random sampling with complete data on body weight categories, weekly PA (Yamax pedometer), ST (family logbook) collected over a regular school/working week during the spring and autumn seasons between 2013 and 2019. Binary logistic regression analyses were used to identify which of parents’ lifestyle indicators were associated with the overweight/obesity of their offspring.

**Results:**

The mother’s overweight/obesity significantly increases her children’s odds of overweight/obesity. Concerning fathers, active participation in organized leisure-time PA and reaching 10,000 steps per day significantly reduce the odds of overweight/obesity in their children and adolescent offspring.

**Conclusions:**

The cumulative effect of parental participation in organized leisure-time PA with their own family-related PA can be a natural means of preventing the development of overweight/obesity in their offspring.

## Introduction

Children and adolescents spend most of their time during childhood at home and in the school environment, where their health, social, and lifestyle-related habits (Mollborn and Lawrence 2018) are formed through education and the application of family and school rules. However, lifestyle habits result in obesity in 5–10% of European children and adolescents on average (Abarca-Gómez et al. 2017; Inchley et al. 2017). There is a considerable cross-national variation in the prevalence of obesity among European children and adolescents. The highest proportion is found in Southern European and Mediterranean countries (Miqueleiz et al. 2016), while a steadily low incidence of obesity among European children and adolescents was reported from North-Western Europe (Abarca-Gómez et al. 2017; Inchley et al. 2017). The rapid increase in childhood and adolescent obesity in Southern and Central and Eastern European countries and Mediterranean countries underlines the fact that these countries have failed to learn effectively from the development of obesity in high-income countries (Inchley et al. 2017; Sigmund et al. 2018a).

Children live together with their parents for a substantial part of their childhood, and parents have a heavy influence on shaping their lifestyle-related habits. Parents therefore act as gatekeepers of their children’s health-related behaviours (Bailey et al. 2015). Similarly to economically developed non-European countries (Garriguet et al. 2017) and Western European countries (Bringolf-Isler et al. 2018), the findings from Central Europe also show a positive relationship between the physical activity (PA) and screen time (ST) of parents and their offspring when the aggregated PA of the family is monitored in an objective way (Sigmundová et al. 2018). However, the associations between the body weight level of parents and their offspring in relation to PA in families in the Central European region are rarely (Erkelenz et al. 2014; Horodyska et al. 2019) and objective monitoring of PA in such studies is even scarcer (Erkelenz et al. 2014). The presented study is thus unique in the region of Central Europe.

Previous studies of parent–child dyads’ behaviour revealed a window of opportunity for increasing PA in overweight/obese Czech preschoolers on weekend days and a significant association between mothers’ excess weight and the overweight/obesity status of their preschool children (Sigmundová et al. 2017). In addition, parental energy balance-related habits (daily step counts (SC) and entertainment (ST)) were linked to the recommended daily SC of their 5–12-year-old children in families with or without an overweight/obese child (Sigmund et al. 2018b). However, no systematic analysis has been conducted of which of the parental lifestyle indicators (daily SC, time of entertainment ST, participation in organized leisure-time PA, and level of body weight) relate to the overweight/obesity of their offspring in a large set of families with children over a wide age range. Therefore, the main aim of this study is to examine the associations of parents’ lifestyle indicators (daily SC, entertainment ST, and participation in organized leisure-time PA) and the level of body weight with overweight/obesity in their offspring in familial aggregation (parent–child dyads and nuclear family triads) in a pedometer-assessed usual weekly routine.

## Methods

### Participants

**Fig. 1:**
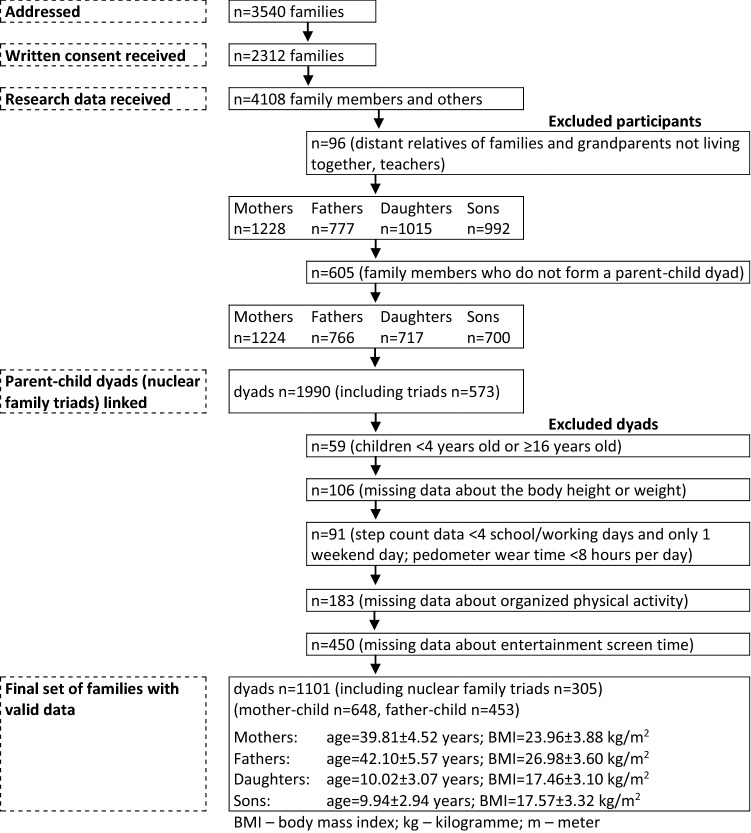
Study flow of participants and basic somatic characteristics of family members (Czechia, 2013–2019)

Participants were recruited by means of two-stage stratified random sampling. In the first stage, nine out of 14 administrative regions, three of each in the lowest, middle, and highest terciles for gross domestic product in the Czechia, were randomly selected. In the second stage of sampling, the selection of kindergarten and primary public schools respected the distribution of the urban–rural population in the Czechia (Ritschelová et al. 2012). Private schools/kindergartens were not addressed, because public schools/kindergartens strongly prevail, and the number of private schools/kindergartens is still negligible in the Czechia. A total of 3540 families, whose children were registered at the selected schools/kindergartens, were addressed in writing with an invitation to participate in cross-sectional study, of which 65.3% agreed to take part in the research (written informed consent received). Participating children and their parents were predominantly white Caucasian (> 98%), which is representative of the ethnic demographics of the Czechia (Ritschelová et al. 2015). A couple and their children who shared living quarters were defined as nuclear family triads (i.e. mother, father, and child together) and participated in the study. If the family has more children, the youngest was included in the study. Family dyads consisted of a mother–child or father–child couple. To be included in the research, at least one dyad (either mother–child or father–child) per family had to provide informed consent. In the initial stage of the study, information meetings were held to describe the process/course of the research. Figure 1 provides a detailed flowchart of the inclusion of the participants in the study.

The analysis included 1101 parent–child dyads (305 of them were nuclear family triads, i.e. mother–father–child) with complete data of family members on weight status, entertainment ST, and ambulatory PA monitored with a Yamax pedometer over a regular school/working week during the spring and autumn seasons between 2013 and 2019 (Fig. 1). The data collection was always arranged to be carried out in a week without school or public holidays.

### Ethics

The study design, format and content of the family logbook, feedback for participants, and the measures and instruments used were approved by the Ethics Committee of the Faculty of Physical Culture review board for families with school-aged children (reference number 50/2012) on 12 December 2012 and for families with preschool children (reference number 57/2014) on 21 December 2014. The ethical principles of the 1964 Helsinki Declaration and its later amendments were adhered to throughout the research. Parental written consent was obtained prior to the start of the data collection.

### Measures and procedures

The schools/kindergartens that agreed to participate in the research received a research design scheme and pedometers so that the children and teachers could become familiar with them. At a subsequent joint meeting of the researchers with parents and school/kindergarten representatives and teachers, all the participants were thoroughly acquainted with the research process, ways of handling the pedometer, and the way of recording data in the family logbook. The parents were given informed consent forms to participate in the research for each family member. Upon receiving written informed consents from family members, the researchers prepared family logbooks. The monitoring of ambulatory PA and sedentary behaviour was launched in the morning of the next meeting with the researchers at the schools.

The children’s and parents’ body height and weight were measured by the parents at home as instructed by the researchers. The anthropometric parameters of all family participants (gender, month and year of birth, body height/weight (with an accuracy of 0.5 cm/kg)) were recorded by the parents in the family logbook before the start of the monitoring of PA and sedentary behaviour. Parental measurement of the body height and weight of their preschool children and schoolchildren at home is a sufficiently valid method to identify overweight/obesity according to the calculated body mass index (BMI) when compared to objective or laboratory measurements (Chai et al. 2019).

The all-day PA of all participating family members was monitored with the same unsealed Yamax Digiwalker SW-200 pedometer (Yamax Corporation, Tokyo, Japan) and quantified by the SC during waking hours. The participants were instructed to wear the pedometer attached to their right hip throughout the whole day for eight consecutive days (during school lessons/paid employment, organized leisure-time PA) except for bathing, showering, and dressing and while performing personal hygiene. Due to time incompleteness and reactivity, the data from the first day of monitoring was not included in the subsequent analyses. Participants were included in the analyses only for at least 6 days with valid PA data containing both weekend days. Every morning after personal hygiene, the parents reset the pedometers and attached them to the right hip of their preadolescent children and to themselves (in adolescent children, they checked their familiarization with the pedometer and its attachment) and recorded the start of the wear time in the family logbook. Every evening, the parents and their offspring took off the pedometers together and wrote down the daily SC and time of the record in the family logbook. They also indicated if they had participated in organized leisure-time PA on that day. Although there are more accurate objective accelerometer-based tools for the measurement of ambulatory PA, counting steps with a pedometer is a simple, unobtrusive, valid, and reliable quantifier of all-day PA for analysis between daily SC and health outcomes (Bassett et al. 2017) across a wide population of children, adolescents (Clemes and Biddle 2013), and adults (Kooiman et al. 2015).

The entertainment ST (sitting/lying watching TV (DVDs, videos) and sitting/lying in front of a PC (notebook, tablet, or smartphone) not for school/work purposes) of all the participants was recorded by the parents together with the children in the family logbook every evening of the monitoring week. The parents were instructed to record the duration of SC with an accuracy of 10 min. The parental proxy reporting of the sedentary behaviour of their offspring showed adequate internal consistency and test–retest reliability (Carson et al. 2017).

### Data management and statistical analysis

All the data management and statistical analyses were conducted using the Statistical Package for the Social Sciences (SPSS) for Windows v.22 software (IBM Corp. Released 2013. Armonk, NY, USA). To maintain the comparability of the prevalence of childhood BMI categories with previous studies (Sigmundová et al. 2017; Sigmund et al. 2018a, b), the BMI categories (underweight, normal weight, overweight, or obese) were derived using age- and gender-specific WHO growth charts (World Health Organization 2007). Overweight or obesity in children is represented by a BMI from the 85th to the 97th percentile of the WHO growth charts (World Health Organization 2007). Overweight and obesity in parents were classified using a BMI from 25 to 29.9 kg/m^2^ and greater than or equal to 30 kg/m^2^, respectively (World Health Organization 2014). Parental underweight was represented by a BMI less than 18 (World Health Organization 2014). The Chi-square (*χ*^2^) test series was used to compare the prevalence of obesity, overweight, normal body weight, and underweight between parents and between children separated by gender. Daily SC values below 1000 or exceeding 30,000 were truncated to these recommended values, respectively (Craig et al. 2013), and included in the analyses. The daily SC recommendation was set at a value of ≥ 13,000/≥ 11,000 steps/day for 5–12-year-old sons/daughters and ≥ 10,000 steps/day for 12–16-year-old adolescents (Tudor-Locke et al. 2011a) and adults (Tudor-Locke et al. 2011b). The daily entertainment ST is the sum of the time spent in front of screen-based devices for non-school/non-work purposes throughout the day. Excessive entertainment ST was defined as more than 120 min per day (Tremblay et al. 2011). Logistic regression models (Enter method) were used to investigate whether excessive body weight of parents and parental lifestyle indicators (achieving the recommended daily SC, non-excessive entertainment daily ST, and participation in organized leisure-time PA) were associated with excessive body weight of their offspring in analyses of parent–child dyads and nuclear family triads (the two sets of analyses separately). The results of the logistic regression analyses were expressed using the odds ratio (OR) and 95% confidence interval (95% CI). The alpha level of significance was set at the minimum value of 0.05.

## Results

**Fig. 2:**
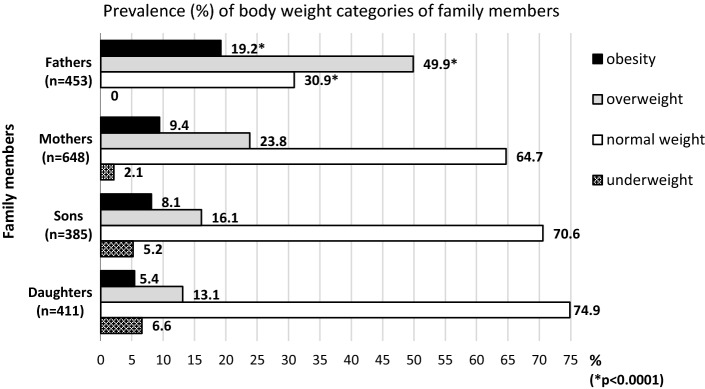
Prevalence of body weight categories of family members (Czechia, 2013–2019). *Statistically significant gender difference between fathers and mothers and between sons and daughters based on the Chi-square tests

Chi-square tests revealed that male participants (fathers/sons) outnumbered female participants (mothers/daughters) in the prevalence of overweight as well as obesity. However, a significantly higher prevalence of overweight (*p* < 0.0001) and obesity (*p* < 0.0001) was only observed in fathers compared with mothers (Fig. 2). Consequently, the lowest proportion of normal body weight among all family participants was found in the group of fathers. The prevalence of underweight in parents was negligible. There was no statistically significant difference between prevalence of underweight and obesity in daughters using the Chi-square test (Fig. 2).

**Fig. 3:**
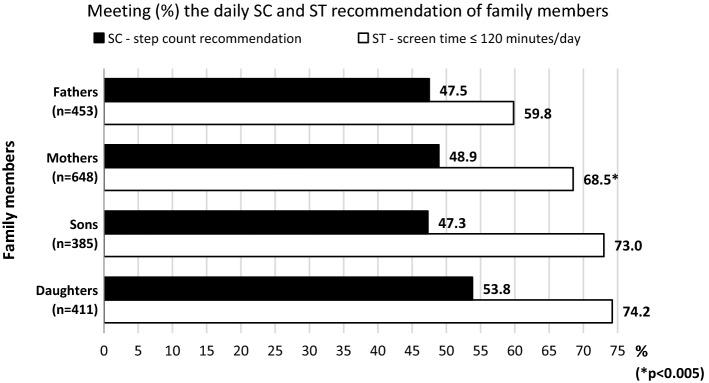
Proportion of meeting the daily SC and ST recommendation of family members (Czechia, 2013–2019). *SC* step counts, *ST* screen time; *statistically significant gender difference between fathers and mothers and between sons and daughters based on the Chi-square tests

The Chi-square tests did not reveal significant differences in the proportion of meeting of the daily SC recommendation between fathers and mothers or between sons and daughters (Fig. 3). Mothers exceeded the daily recommended 120-minute entertainment ST limit less often than fathers. There was no significant difference between daughters and sons in exceeding the daily recommended entertainment ST time (Fig. 3).

While none of the lifestyle correlates of the child/adolescent participants (achieving the recommended daily SC, > 2 h per day of entertainment ST, or participation in organized leisure-time PA) was significantly associated with their overweight or obesity, maternal body weight status and PA and the father’s participation in organized PA were significantly associated with the overweight/obesity of their offspring (Table 1). Not surprisingly, excessive maternal body weight was significantly (*p* < 0.001) associated with higher odds of the prevalence of overweight/obesity in their offspring in the parent–child dyads, as well as in the nuclear family triads. Conversely, the fathers’ achievement of 10,000 steps per day in the analyses of the parent–child dyads and engagement in organized leisure-time PA at least once a week in the fathers in the nuclear family triads were significantly (*p* < 0.05) associated with lower odds of overweight/obesity in their offspring. The mothers’ participation in organized leisure-time PA and their own PA of at least 10,000 SC per day also reduced the odds of overweight/obesity in their children, but this relationship was not significant (Table 1).

**Table 1:** Impact of parents’ indicators on odds of excessive body weight in their offspring (Czechia 2013–2019)

	Excessive body weight (overweight/obesity) in their offspring					
	Family nucleus triads		Parent–child dyads			
	A—Mother B—Father		Mother in model		Father in model	
	OR	95% CI	OR	95% CI	OR	95% CI
*Parent:*						
Weight status						
Non-overweight	Ref.		Ref.		Ref.	
Overweight/obesity	A 2.85**	1.56–5.20	2.60**	1.74–3.89	1.58	0.92–2.71
	B 1.09	0.56–2.11				
Daily SC						
< 10,000 steps/day	Ref.		Ref.		Ref.	
≥ 10,000 steps/day	A 0.84	0.46–1.57	0.88	0.59–1.31	0.62*	0.39–0.99
	B 0.81	0.44–1.48				
Screen time						
> 120 min per day	Ref.		Ref.		Ref.	
≤ 120 min per day	A 0.69	0.37–1.30	0.72	0.48–1.09	1.09	0.69–1.73
	B 1.27	0.68–2.38				
Organized PA						
0 times per week	Ref.		Ref.		Ref.	
1 times per week	A 0.93	0.45–1.91	0.72	0.47–1.10	0.58*	0.35–0.96
	B 0.45*	0.21–0.95				

The binary logistic regression models presented in this table were adjusted for gender and age category of children; *SC* step counts (daily SC recommendations: ≥ 13,000/≥ 11,000 steps/day for boys/girls and ≥ 10,000 steps/day for 12–16-year-old adolescents), *PA* physical activity, *OR* odds ratio, *95% CI* confidence interval; *Ref*. reference group

The statistical significance is expressed as *p < 0.05; **p < 0.001

## Discussion

The present study identifies relationships between excessive body weight in parents and their offspring under the spotlight of family PA/ST behaviour patterns. Family-aggregated pedometer-based PA and proxy-reported entertainment ST patterns were revealed in an extensive set of families with children aged four to 16 years under daily life conditions.

Although the observed prevalence of overweight/obesity in Czech children and adolescents is not as high as in Southern Europe or Mediterranean countries (Inchley et al. 2017) or among US children and adolescents (Skinner et al. 2018), it is worrying that the trend in the prevalence of overweight/obesity has not reached its plateau yet (Sigmund et al. 2018a). In particular, the rates of obese adolescents are still increasing (Sigmund et al. 2019). There is no doubt that overweight and obesity pose many health risks not only in adulthood (Xu et al. 2018) but already in early childhood (Sahoo et al. 2015), and that excessive weight gain in childhood and adolescence is likely to lead to lifelong overweight and obesity (Singh et al. 2008). Weight loss and post-weight loss maintenance are difficult to achieve, and the long-term application of short-term proven multi-component obesity-reducing interventions tends to fail to work in real life (Beets et al. 2019). Thus, it is still desirable to seek indicators that can potentially reduce the chances of childhood obesity occurring in the conditions and environments of usual daily life. While state schools provide universal access to a structured environment that is, in most instances, health-promoting, extracurricular, and family settings do not guarantee the health-promoting stimulation of children and adolescents (Beets et al. 2019).

Unlike other studies (Francesquet et al. 2019; Jääskeläinen et al. 2011), we found a significantly higher odds ratio of being overweight/obese in offspring only in the case of their mothers’ overweight/obesity. The closer relationship between overweight/obesity of mothers and their offspring, compared with father–child dyads, may be explained by the fact that children (especially preschool and younger school age) are more likely to adopt patterns of maternal behaviour (including ST) than fathers’ patterns, because the household is mainly taken care of by mothers (Sigmundová et al. 2017). Moreover, the strong genetic linkage between the development of excessive body weight of children and their parents is confirmed by the results of a 16-year follow-up study, which show that overweight parents pose a high risk of overweight/obesity in children at birth and also at the age of 16 (Jääskeläinen et al. 2011).

This study attempted to bridge the research gap in Central European countries using the family dyad approach to investigate the relationships between parental lifestyle indicators and the excess body weight of their offspring. Unlike other studies (Bringolf-Isler et al. 2018; Francesquet et al. 2019; Garriguet et al. 2017; Jääskeläinen et al. 2011), we analysed indicators of active lifestyle and overweight of parents with the overweight of their offspring together. Our results show that greater PA of parents (≥ 10,000 SC per day) and their active participation in organized leisure-time PA (≥ 1 times per week) is linked to a lower odds ratio of overweight/obesity in their offspring. This was significant only in the case of father–child associations, though. While mothers probably take more care of their household than fathers, fathers have more time left to organize their children’s leisure activities and play sports with their children than mothers do. Therefore, the regular joint sport of fathers with children may result in a lower chance of childhood obesity compared to less active fathers. However, other studies have not found different associations between parent–child BMI classified according to the different frequency of moderate-to-vigorous PA per week (Liu et al. 2013). A lower prevalence of overweight/obesity in children was observed in parents who were actively involved in sports, compared with inactive parents despite the lack of association between parent–child PA (Erkelenz et al. 2014). Parental support for PA in children seems to be very important for the implementation of children’s PA (Solomon-Moore et al. 2018). In our case, more active fathers may be more likely to facilitate participation of their offspring in sports (and physical activity, in general) than less active fathers.

### Limitations, strengths, and future studies

The present results and conclusions drawn should always be interpreted in the spotlight of existing methodological limitations. Since pedometers are limited in detecting the intensity of PA, the total daily SC may be reduced, especially in the case of participation in organized sports characterized by performing high-intensity PA. On the other hand, the total daily SC detected by a spring-suspended waist-mounted Yamax pedometer could be reduced by insensitivity during slow walking (Bassett et al. 2017). Although none of the family participants was informed about the SC recommendation for the given age category for the variables that were monitored, there is always the risk of bias resulting from social desirability (Clemes and Biddle 2013) or the degree of parental diligence in recording data in the family logbook. Attempts have been made to at least see if there are differences in the recorded SC/ST values in the family logbook according to the gender of the parent making the record, and it is reassuring that the recorder’s gender does not affect the values of the SC/ST variables. Furthermore, ST multitasking [simultaneous use of multiple screen devices or different activities on one screen device—(van der Schuur et al. 2015)] was not studied in detail. However, the participants were instructed to record the entertainment ST on the family record sheet if this was the main way in which this time was used. Nevertheless, the aim of this study was not to determine the total daily SC/ST as accurately as possible, but to analyse the relationship between overweight/obesity and PA-related behaviour in family aggregation. The associations of parents’ weight status with weight status of their children could have been influenced by other factors of lifestyle such as, eating habits or alcohol consumption, which were not monitored in the present study. Future studies should therefore account for these factors too. The age categories of parents did not play a significant role in the prevalence of overweight/obesity or the relationship to prevalence of overweight/obesity of their children.

The wide age range of the participants, the same well-trained team of researchers, and the relatively stringent inclusion criteria (number of days and daily monitoring periods) can be considered as strengths of the study. In line with serious recommendations (Tremblay et al. 2016), a new space opens up to find correlates of excessive body weight in children and adolescents during the 24-h monitoring of movement behaviour within the family lifestyle. Given the trend of the age of both mothers and first-time mothers in the Czechia increasing between 2001 and 2017 (Frelich 2018), it would be interesting to analyse whether the increasing age difference between parents and their offspring influences the relationship between the level of body weight of parents and their children or parent–child PA, as studied, for example, in Germany (Bringolf-Isler et al. 2018).

## Conclusions

Despite the significantly higher odds of overweight/obesity in children in the case of maternal overweight/obesity, the active involvement of fathers in organized leisure-time PA considerably reduces the likelihood of excessive body weight in their offspring. The cumulative effect of parental participation in organized leisure-time PA with their own family-related PA can represent a natural means of preventing excessive body weight in their own offspring.
